# Factors Involved in the Perception of Enamel Dyschromic Lesions—A Questionnaire-Based Study

**DOI:** 10.3390/ijerph19020900

**Published:** 2022-01-14

**Authors:** Cristina Gasparik, Horațiu Alexandru Colosi, Bianca Elena Varvara, Alexandru Grațian Grecu, Alexandra Iulia Aghiorghiesei, Anca Ștefania Mesaroș, Amalia Mazilu (Moldovan), Diana Dudea

**Affiliations:** 1 Department of Prosthetic Dentistry and Dental Materials, Iuliu Hatieganu University of Medicine and Pharmacy, 400006 Cluj-Napoca, Romania; gasparik.cristina@umfcluj.ro (C.G.); bianca.varvara@yahoo.com (B.E.V.); alexgreeck@yahoo.com (A.G.G.); ada_irimie@yahoo.com (A.I.A.); ancames@yahoo.com (A.Ș.M.); ddudea@umfcluj.ro (D.D.); 2 Department of Medical Education, Division of Medical Informatics and Biostatistics, Iuliu Hatieganu University of Medicine and Pharmacy, 400349 Cluj-Napoca, Romania; 3 Physics and Chemistry Department, Technical University of Cluj-Napoca, 400114 Cluj-Napoca, Romania; amalia.mazilu@gmail.com

**Keywords:** dyschromic lesion, perception, color, esthetics

## Abstract

Dyschromic lesions (DLs) of tooth enamel are common disorders, with multiple etiologies and various clinical forms, that raise public health concerns. This study aimed to determine the factors that influence the perception of DLs and to assess the perceived need for dental treatment in various clinical cases. A paper-based questionnaire with attached images of frontal teeth exhibiting different DLs was distributed to patients, dental students, and dentists. A total of 383 volunteers participated in this study, and their answers were statistically described and analyzed. This study found that in cases with multiple, well-demarcated areas of opacities associated with diffuse opacities on neighboring teeth, most respondents noticed and reported only the most severe lesions, disregarding the minor ones. The contrast of the lesion with the color of the substrate influenced the overall perception and a significant correlation between these two variables was found. However, the color of the DLs did not significantly impact the overall perception of the lesions. A higher overall perception of DLs was significantly correlated with a favorable opinion regarding the need for treatment. Furthermore, gender and medical background were significantly associated with the overall perception of DLs.

## 1. Introduction

Perception and self-perception of body image are multidimensional and influenced by social, cultural, behavioral, and psychological factors [1,2]. In addition, the esthetics of the lower third of the face plays a significant role in interpersonal interactions, as the interlocutor often focuses attention on this region during communication. Several attributes are considered critical when judging the esthetics of the smile: size, shape, color, texture, alignment, symmetry, position, visibility of the teeth, aspect of the gums, and the appearance of the lips [3]. Nevertheless, their impact may be variable depending on the particularities of each smile.

The perception of an abnormal dental color often motivates patients to seek dental treatment [4,5]. A questionnaire-based study reported that adolescents associate their tooth color with the subjective perception of oral health [6]. Furthermore, the study of Samorodnitzky-Naveh et al. [7], evaluating the perception of tooth color, conducted on 407 subjects, concluded that 37.3% of them were dissatisfied with their dental color.

Considering the extension along the dental arches, tooth discoloration can be localized, involving one tooth or arch-segment, or generalized when the entire dentition is affected. When the relation of the staining agent with the tooth surface is concerned, extrinsic and intrinsic discolorations are described [3]. Generally, extrinsic discoloration is produced by stains deposited on tooth surfaces. In contrast, intrinsic dyschromic conditions are caused by modifications of the tooth structure and composition, which occur before or after the dental eruption. As result, the discoloration can be a uniform alteration of the initial dental color, in the case of pulp pathology and endodontic treatments.

In other circumstances, these lesions appear as single or multiple-colored areas on the dental surface, with variable etiology and clinical appearance from bright white opacities to regions with different extensions and colorations [3].

White spot lesions (WSLs) is a common term often used to describe non-cavitated lesions. However, this term suggests only the color aspect without considering the etiology of the lesion [8]. WSLs can have different clinical forms, such as well-demarcated opacities, with varying degrees of severity, to diffuse opacities, with less visible boundaries.

Dental fluorosis is a developmental defect of the tooth structure that occurs due to the excessive ingestion of fluoride. Very mild and mild fluorosis is characterized by diffuse white spots or lines on the tooth surfaces. In moderate and severe fluorosis, the enamel color and texture are affected, having chalky white stains, pits, and surface defects that often become stained over time [3].

Enamel hypocalcification is a condition characterized by white opacities on the dental surfaces, caused by alterations in the mineralization process during enamel formation [3].

Traumatic hypomineralization occurs after the trauma of primary teeth and usually appears asymmetrically as white patches in the incisal third of permanent anterior teeth [9].

The molar-incisor hypomineralization (MIH) has a systemic origin and is characterized by white demarcated enamel opacities affecting the first permanent molar and incisors. These lesions are usually located in the thickness of the enamel, closer to the dentin-enamel junction. The color aspect of these lesions varies from white-creamy to yellow-brown opacities [9].

Demineralized lesions are caused by poor oral hygiene and are frequently associated with orthodontic treatment. Demineralized areas have a chalky white aspect, usually located around orthodontic brackets or along the gingival margin, frequently associated with gingival inflammation [9].

Several studies investigated the esthetic impact of enamel opacities and concluded that these lesions were less favorably perceived than other dental conditions [10,11,12,13]. Nevertheless, it is not clear which characteristics of the lesions significantly influence their perception and what determines the observers to judge differently the areas of discolorations that may affect the dental surfaces.

Although many studies focus on the etiology and treatment of localized dyschromic areas on dental surfaces, little is known about how individuals perceive these lesions and if their presence and appearance motivate patients to seek dental treatment. Therefore, our study aimed to assess the esthetic importance of WSLs and other dyschromic lesions (DLs) through a paper-based questionnaire. The objectives of the study were to determine how individuals perceive DLs with different localizations, degrees of severity, color, surface texture, and to assess their options regarding the opportunity of dental treatment in each case; to determine whether an association may exist between the characteristics mentioned above of dyschromic lesions and the age, gender, or medical background of the responders.

The null hypotheses were: 1. The perception of dyschromic lesions (DLs) was not dependent on the location, the extension, or color of the lesion on the dental surface; 2. The option to treat the DLs was not dependent on the perception of the lesion; 3. There was no association between age, gender, medical background, and the perception, extension, or option for treatment of DLs.

## 2. Materials and Methods

### 2.1. The Questionnaire

**Figure 1:**
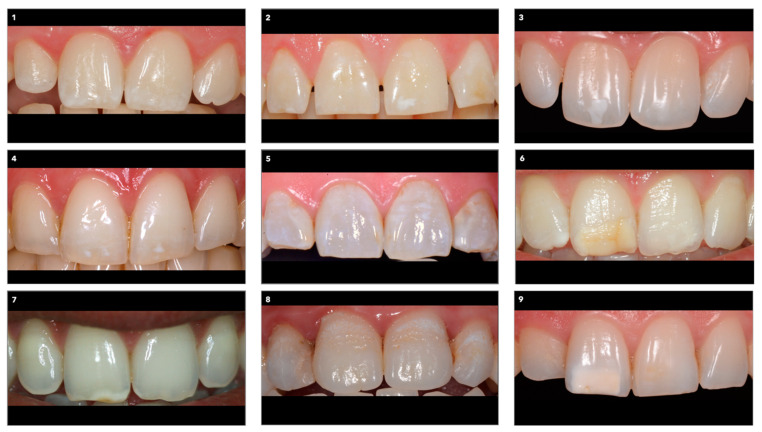
The set of images linked to the questionnaire.

The paper-based questionnaire was approved by the Ethics Committee of the Iuliu Hatieganu University of Medicine and Pharmacy Cluj-Napoca, Romania (162/18.05.2021). It contained demographic information (age, gender, area of origin, nationality, education) and nine questions with single or multiple-choice answers. The questions were linked to nine images of dental arches displaying the upper central and lateral incisors; the lips were retracted, showing the entire labial surfaces of the incisors. The images were arranged randomly on an A4 photographic paper (Figure 1) and were selected to include dyschromic conditions, such as single white spot lesions with different severity levels and locations on the labial surface; single yellow-orange lesions with different severities and locations on the labial surface; multiple white spot lesions located in the incisal area; multiple white or/and yellowish lesions, covering the whole labial surfaces of the incisors. The dyschromic areas affected one tooth, several teeth, or the entire incisor group displayed in the image.

**Figure A1:**
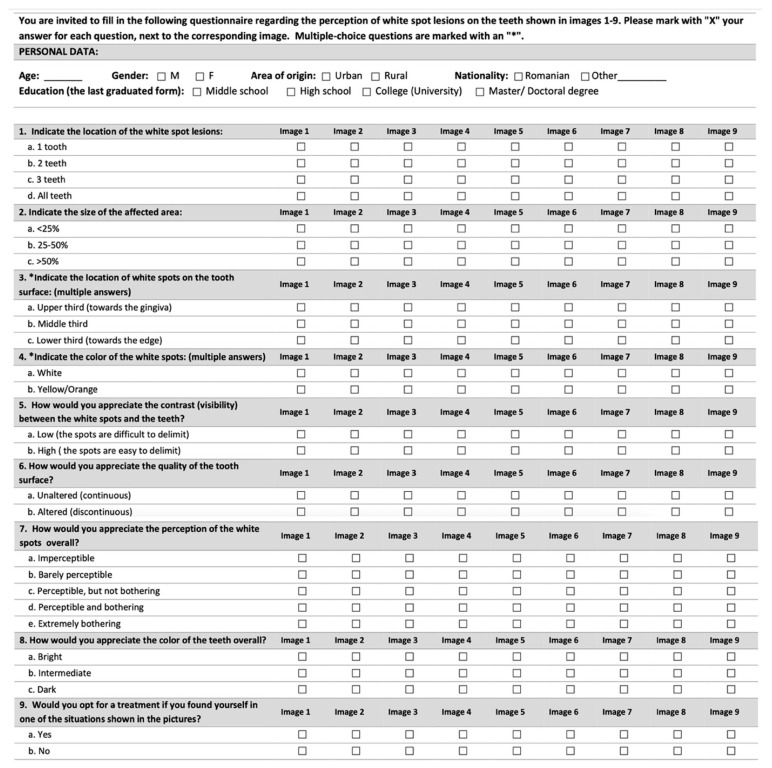
The structure of the questionnaire.

The questions investigated the perception of location, color, and size of the lesions, the number of affected teeth, the overall color and surface texture of the teeth, as well as the visibility of the dyschromic areas; the perceived opportunity for treatment was also inquired about (Appendix A Figure A1). Every question formulated in the questionnaire concerned each of the nine images.

The questionnaire was initially designed in Romanian and subsequently translated into English and French by faculty teaching Esthetic Dentistry to dental students enrolled in English and French sections of the Iuliu Hatieganu University of Medicine and Pharmacy Cluj-Napoca. The questionnaire was applied in all three languages. The questionnaire was previously validated in a pilot study on 30 dental students, 10 for each language.

### 2.2. The Participants

The questionnaire and its associated images were distributed between January and June 2021 to patients attending private dental offices in Cluj-Napoca, to dentists with different experience levels (residents, specialists, teaching staff), and to dental students of the Iuliu Hatieganu University of Medicine and Pharmacy Cluj-Napoca. Participation was voluntary, and individually identifiable information, such as names, addresses, or personal codes, was not collected. Each participant was informed about the aim of the study, and the questionnaire structure was explained by the person distributing each questionnaire. Questionnaires with incomplete answers were excluded.

### 2.3. Statistical Analyses

Answers to the questionnaire were recorded consecutively in a database and were statistically described and analyzed using Microsoft Excel 2010 (Microsoft Corporation, Redmond, WA, USA) and IBM SPSS Statistics v.25 (IBM, Armonk, NY, USA). The hypotheses were tested using Pearson’s chi-square tests, and correlations were assessed using Kendall’s tau b correlation coefficients. The significance level was set to α = 0.05.

## 3. Results

A total of 383 questionnaires with complete answers were analyzed in the study. Demographic information about the study participants is presented in Table 1.

### 3.1. Location of the DLs (WSLs and Color-Affected Areas) of the Teeth

Most of the participants indicated one (images 3, 7, and 9) or two teeth (images 1, 2, 4, and 6) as being affected by DLs, except for images 5 and 8, for which the DLs were perceived as involving all the four frontal teeth (Figure 2).

**Figure 3:**
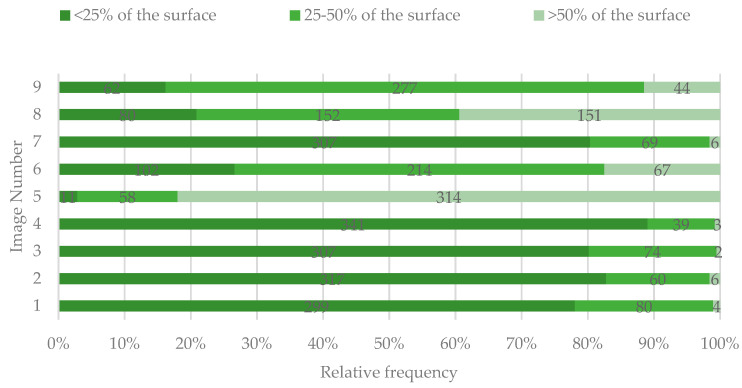
Percentage of the buccal tooth surface identified by study participants as affected by DLs.

**Figure 4:**
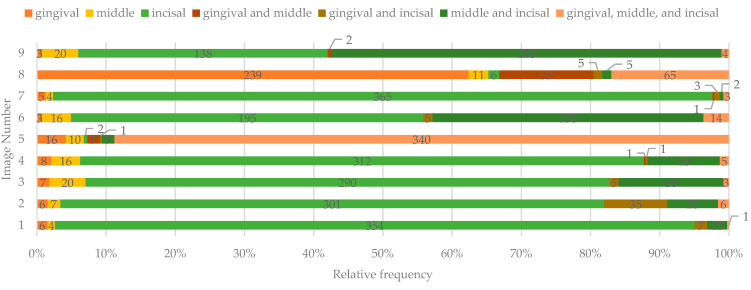
The affected third of the buccal tooth surface identified by study participants.

In images 1–4 and 7, the majority of the participants indicated that the lesions affected less than 25% of the tooth surface. For image 5, 81.98% of the respondents considered that the lesions affected more than 50% of the tooth surface. For images 6 and 9, most participants indicated that the tooth surface was affected between 25–50%. Furthermore, there was a consensus regarding the tooth area involved for these images, the majority indicating the middle and incisal areas. For image 5, most respondents indicated that all three areas were involved, while for image 8, most respondents considered that only the gingival area was affected (Figure 3 and Figure 4).

### 3.2. Color, Contrast, and Surface Texture of the DLs

**Figure 5:**
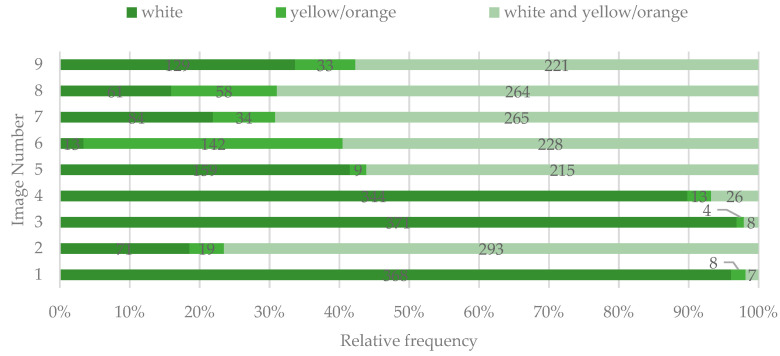
The perceived color of the lesions as having DLs identified by study participants.

The color of the lesions was judged as white by the majority of the respondents only in images 1, 3, and 4. For the rest, the color was considered as both white and yellow/orange (Figure 5).

**Figure 6:**
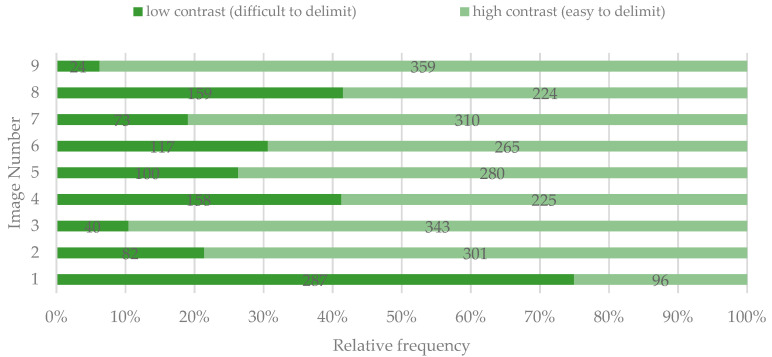
Perceived contrast (visibility) of the spots identified by study participants.

Except for image 1, most participants considered that there was a high contrast between the lesions and the surrounding tooth surface (Figure 6).

A weak positive correlation was found between the color of the lesion and the contrast (visibility) of the DLs, but only for images 1 (χ^2^(2, *n* = 383) = 6.743, *p* = 0.034; τ_b_ = 0.131, *p* = 0.048) and 8 (χ^2^(2, *n* = 383) = 13.326, *p* = 0.001; τ_b_ = 0.158, *p* = 0.002). The white lesion in image 1 was difficult to delimit, while the white and yellow/orange lesions in image 8 were easy to delimit.

**Figure 7:**
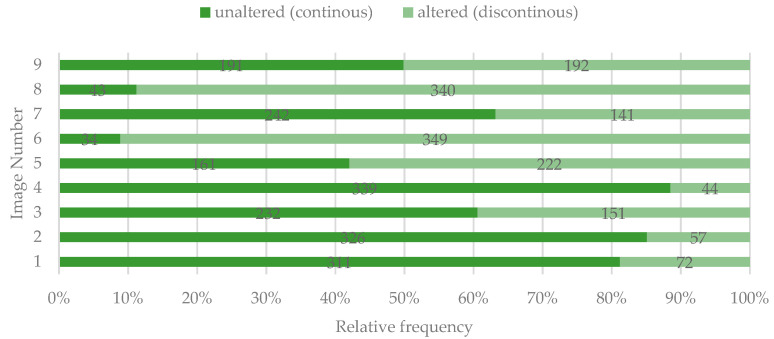
Perceived quality of the tooth surface identified by study participants.

For images 5, 6, 8, and 9, the surface was considered as being altered by most of the participants (Figure 7).

### 3.3. The Overall Perception of the DLs

The least bothersome were the situations presented in images 1, 2, and 4 (lesions with lower contrast, severity, and incisal location). In contrast, the most unpleasant were considered the ones in images 5, 6, 8, and 9 (lesion with high contrast, extension, and severity) (Figure 8).

The overall perception of the DLs was correlated with the color of the lesion only for images 2 (χ^2^(6, *n* = 383) = 17.110, *p* = 0.009; τ_b_ = 0.168, *p* = 0.000) and 8 (χ^2^(8, *n* = 383) = 27.217, *p* = 0.001; τ_b_ = 0.155, *p* = 0.001). Yellow/orange lesions were perceived as more bothersome than white lesions. However, only a weak correlation was found between these two variables.

Nevertheless, the overall perception was correlated with the visibility (contrast) of the lesion in all the situations presented in images 1–9. Although only a weak correlation was found between overall perception and visibility, the lesions with high contrast were perceived as more problematic for the respondents (Table 2).

### 3.4. Opinion Regarding the Treatment Need for the Dyschromic Areas

**Figure 9:**
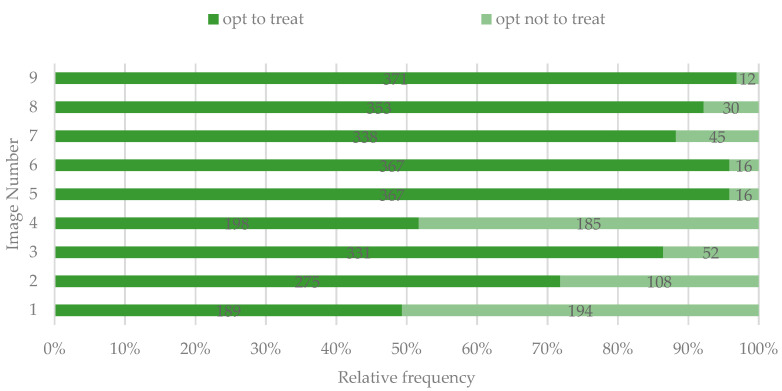
Option of study participants to treat the DLs.

Regardless of the type of the lesions, most participants would request treatment of the DLs if found in a situation presented in the proposed images (Figure 9). The expression of treatment need for different DLs was correlated with the overall perception of the lesion for all the situations presented, except for image 9 (Table 3).

No statistically significant association between age and overall perception of the DLs was found, regardless of the situations presented in images 1–9 (p > 0.05). 

**Figure 10:**
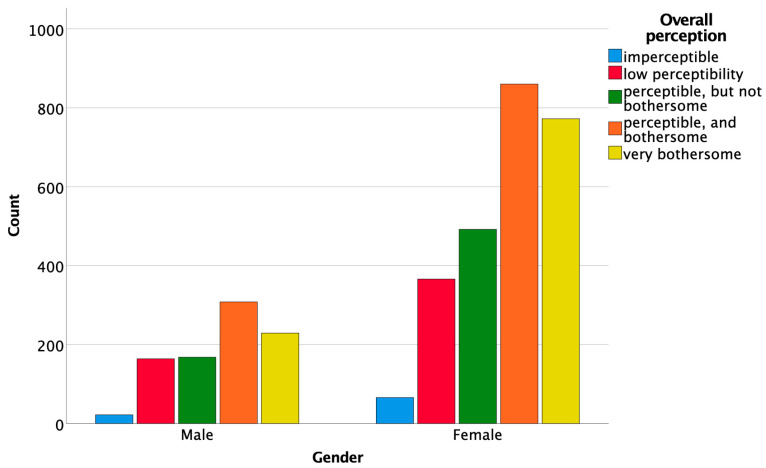
Association between gender and the overall perception of the DLs.

**Figure 11:**
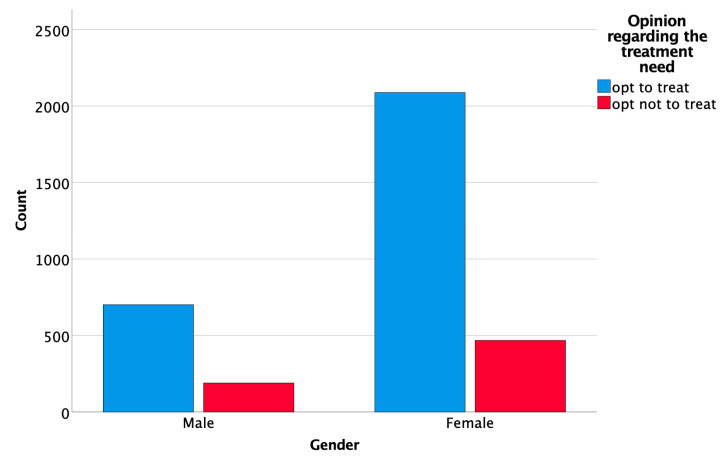
Association between gender and opinion regarding the treatment need.

Female respondents exhibited a more frequent with a more bothersome overall perception of DLs, compared to male respondents (χ^2^(4, *N* = 3447) = 12.041, *p* = 0.017) and offered more favorable answers regarding the treatment need they perceived (χ^2^(1, *N* = 3447) = 3.887, *p* = 0.049) (Figure 10 and Figure 11). However, no association was found between gender and the contrast of the DLs (χ^2^(1, *N* = 3447) = 1.065, *p* = 0.302).

In addition, there was no statistically significant association between medical background and the perception of DLs (χ^2^(24, *N* = 3447) = 35.221, *p* = 0.065), or between medical background and the perceived contrast of DLs (χ^2^(6, *N* = 3447) = 3.615, *p* = 0.729). Nevertheless, the overall intention to request treatment for DLs was significantly associated with the medical background and specialty of the respondents (χ^2^(6, *N* = 3447) = 22.100), *p* = 0.001), more lay-persons and dentistry students, but also more prosthodontists preferring to request treatment if found in situations like the ones presented in images 1–9, compared to specialists in general dentistry, orthodontics or maxillofacial surgery.

## 4. Discussion

Dyschromic areas affecting the enamel are common clinical disorders with multiple etiologies and various appearances that raise public health concerns. 

Several studies reported that severe fluorosis was associated with a lower oral health-related quality of life (OHRQoL) [13,14]. De Amorim et al. reported that 74.1% of permanent teeth display discoloration and hypoplasia after traumatic dental injuries in primary teeth [15]. Freitas Fernandes et al. stated that the prevalence of MIH in a Brazilian schoolchildren population was 10.8% [16]. In a study evaluating the OHRQoL after minimally invasive treatment of MIH, Hasmun et al. reported an improvement of the self-reported OHRQoL, reducing the visibility of the lesion and having a positive impact on the children’s wellbeing [17]. Julien et al. quantified the prevalence of WSLs on the anterior teeth after orthodontic treatment and concluded that 23.4% of the patients developed at least one WSL during the therapy [18]. Significantly higher values (68.4%) were reported in the meta-analysis conducted by Sundararaj et al. on the prevalence of WSLs during fixed orthodontic appliance treatment [19]. 

Several studies conducted in countries with extensive water fluoridation reported that even mild dental fluorosis represents an esthetic concern for individuals [10,11,12,20]. In addition, the findings of Sigurjóns et al. revealed that very mild and mild fluorosis could be noticeable and raise concerns for parents whose children were affected by these lesions [21]. On the contrary, Browne et al. concluded in their questionnaire-based study that low levels of enamel fluorosis were judged similarly to no fluorosis when evaluating the esthetic impact of these lesions in a group of adolescents [1].

Our study aimed to assess the esthetic impact of dyschromic enamel lesions, considering different characteristics such as number, color, and texture of the lesions, the contrast with the adjacent dental surface, in an attempt to identify the most significant factors involved in the perception of such lesions.

The first null hypothesis that the perception of the DLs was not dependent on the location, the extension, or color of the lesion on the dental surface was rejected. We found that study participants regarded and interpreted differently the number and the location of the DLs presented in the images attached to the questionnaire. 

In cases with multiple well-demarcated areas of opacities associated with diffuse opacities on neighboring teeth, most of the respondents noticed and reported the most severe lesion, disregarding the minor lesions. This dominant effect of the severe lesion can be observed in images 2, 3, 6, and 9. Similar results were reported by Elwood et al. [10], who investigated the esthetic impact of demarcated opacities. They concluded that large or medium well-contoured enamel opacities and moderate to severe hypomineralization have a more significant impact on both dentists’ and patients’ perception than small, demarcated opacities and mild diffuse hypocalcification. Kavand et al. reported that the number of tooth areas affected by fluorosis was negatively associated with both parents’ and adolescents’ level of satisfaction regarding dental appearance [22]. In addition, we also found that the lesions that affected the central incisors were easily perceived. 

The color of the lesions was easily recognized by the participants; however, when lesions with different colors were in the same image, they were sometimes judged differently by the responders. For instance, the lesions in images 2, 6, 7, 8, and 9 presented both white and yellow/orange discolorations. However, some study participants judged the lesions as only white or only yellow/orange. This result could also be explained by the dominance effect of the most important color modification.

The contrast of the lesion with the surrounding tooth surface was judged as high by most of the study participants in all images, except for image 1. Nevertheless, for images 4, 5, 6, and 8, a considerable number of participants judged the lesions as having low contrast. The lesions in these images have a color closer to the adjacent tooth surface; therefore, their perception might have been influenced by the surroundings. Moreover, the contrast of the lesion influenced the overall perception, and a significant correlation between contrast and perception was found for each of the images attached to the questionnaire.

The color of the DLs did not seem to significantly impact the overall perception of the lesions since only a weak correlation was found between these two variables and only for two of the proposed images. However, the yellow/orange lesions were considered more disturbing factors in the perception of the lesions. Da Silva et al. also concluded that yellowish opacities with tooth surface alteration negatively influenced the perception and social judgments of the responders [23].

The second null hypothesis was also rejected. The overall perception of the DLs influenced the intention to request treatment. When the lesions were perceived as bothersome, most responders would ask for treatment. These results are in accordance with previous research, which concluded that the desire for treatment matched closely with an unacceptable appearance of teeth [24]. However, interestingly, Edwards et al. reported that increasing the viewing distance of the lesions lessened the esthetic impact, leading to fewer participants who claimed the treatment was necessary [25]. Another study reported that enlarged pictures of the oral cavity significantly affected the perception, suggesting an exaggerated effect over the esthetic impact of the lesions [26]. In the present study, we have used images of teeth with the same magnification, assuring, from this point of view, uniform perception and evaluation of the displayed incisors group. 

The perception of dental esthetics varies among individuals and is dependent on different factors such as gender and age [27,28]. In our study, no significant correlation was found between age groups and the overall perception of the DLs. Therefore, the third null hypothesis was partly rejected. However, the number of study participants was not uniformly distributed within the age groups, which could have influenced the results.

On the other hand, gender was significantly associated with the overall perception of the lesions. Women tended to be more critical regarding the perception of the lesions and would request treatment more often than men. This finding is in agreement with the results of previous studies that investigated the differences in color perception between men and women [29,30,31].

This study also found that most non-professionals and dental students would request treatment of the DLs if found in any of the situations presented in the questionnaire. The difference in observed versus expected responses between lay-persons, dentistry students, and prosthodontists compared to other specialties of dentistry might be explained by a higher focus on removing the esthetic effects of DLs in the former three categories, compared to other specialties of dentistry, who might be less involved in esthetic treatments aiming to mask dyschromic conditions. In a study by Wong et al. [28], the authors concluded that dentists’ perception of the need for treatment was correlated with the type, size, and color of the lesion, but also with the age of the dentist.

In the current study, the responders were not asked to motivate their answers, and the interpretations of the data relied only on descriptive results and statistical analysis. Other limitations of the current study derive from the fact that the studied sample was not balanced against the general population in respect of its male/female ratio, the proportions of different age groups, or the proportions of patients, dentistry students, and of the different specialties of dental medicine represented in the sample.

## 5. Conclusions

Within the limitations of the present study, it was concluded that:

1. Perception of DLs is influenced by the number of affected teeth, the severity of the lesion, and their contrast with the surrounding dental surface; however, the color of the lesions did not seem to play a significant role in the esthetic perception.
2. The opinion regarding the need for treatment of DLs correlated significantly with the overall perception of the DLs.
3. Gender and medical background significantly influenced the overall perception of DLs.

## Figures and Tables

**Figure 2:**
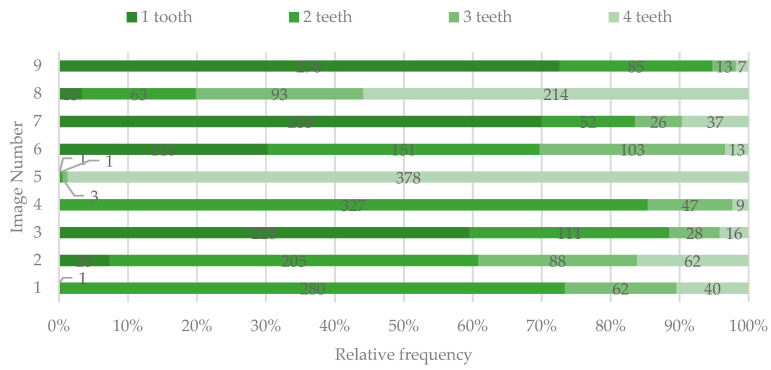
The number of teeth identified by study participants as having dyschromic lesions (DLs).

**Figure 8:**
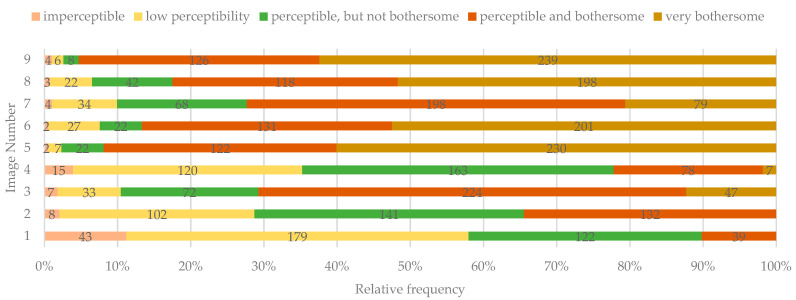
The overall perception of the lesions identified by study participants.

**Table 1:** Demographic characteristics of the studied group (*n* = 383).

Age							
<20y	20–29y	30–39		40–49y	50–59y	>60y	
20.37% (*n* = 78)	63.19% (*n* = 242)	7.83% (*n* = 30)		6.79% (*n* = 26)	1.04% (*n* = 4)	0.78% (*n* = 3)	
Gender		Area of origin			Nationality		
Males	Females	Urban		Rural	Romanian	Other	
25.85% (*n* = 99)	74.15% (*n* = 284)	78.59% (*n* = 301)		21.41% (*n* = 82)	79.63% (*n* = 305)	20.36% (*n* = 78)	
Education							
Middle school		High school		College/University		Doctoral/Masters’ degree	
1.57% (*n* = 6)		66.58% (*n* = 255)		25.33% (*n* = 97)		6.52% (*n* = 25)	
Profession							
Non-professionals				Dentists			
77.55% (*n* = 297)				22.45% (*n* = 86)			
Dental students	Patients	General dentists	Prostho-dontists	Ortho-dontists	Endo-dontists	Oral surgeons	Perio-dontists
36.81% (*n* = 141)	40.74% (*n* = 156)	11.63% (*n* = 10)	47.67% (*n* = 41)	20.93% (*n* = 18)	11.63% (*n* = 10)	4.65% (*n* = 4)	3.49% (*n* = 3)

**Table 2:** Correlation between contrast and overall perceptibility of the dyschromic lesions (DLs).

	Imperceptible		Low Perceptibility		Perceptible But Not Bothersome		Perceptible and Bothersome		Very Bothersome		
	Low Contrast	High Contrast	Low Contrast	High Contrast	Low Contrast	High Contrast	Low Contrast	High Contrast	Low Contrast	High Contrast	Sign.
Image 1	35	8	157	22	81	41	14	25	0	0	χ^2^(3, *N* = 383) = 52.889, *p* = 0.000; τ_b_ = 0.296, *p* = 0.000
Image 2	5	3	33	69	28	113	16	116	0	0	χ^2^(3, *N* = 383) = 22.257, *p* = 0.000; τ_b_ = 0.207, *p* = 0.000
Image 3	4	3	12	21	14	58	7	217	3	44	χ^2^(4, *N* = 383) = 59.919, *p* = 0.000; τ_b_ = 0.297, *p* = 0.000
Image 4	10	5	64	56	65	98	15	63	4	3	χ^2^(4, *N* = 383) = 27.689, *p* = 0.000; τ_b_ = 0.225, *p* = 0.000
Image 5	0	2	3	4	14	8	37	85	46	184	χ^2^(4, *N* = 383) = 23.359, *p* = 0.000; τ_b_ = 0.187, *p* = 0.000
Image 6	1	1	13	14	12	10	45	86	46	155	χ^2^(4, *N* = 383) = 16.726, *p* = 0.002; τ_b_ = 0.188, *p* = 0.000
Image 7	2	2	19	15	17	51	24	174	11	68	χ^2^(4, *N* = 383) = 41.450, *p* = 0.000; τ_b_ = 0.222, *p* = 0.000
Image 8	2	1	15	7	25	17	51	67	66	132	χ^2^(4, *N* = 383) = 18.435, *p* = 0.001; τ_b_ = 0.194, *p* = 0.000
Image 9	2	2	0	6	1	7	11	115	10	229	χ^2^(4, *N* = 383) = 17.022, *p* = 0.002; τ_b_ = 0.118, *p* = 0.040

**Table 3:** Correlation between the expression of treatment need and overall perception of the DLs.

	Imperceptible		Low Perceptibility		Perceptible But Not Bothersome		Perceptible and Bothersome		Very Bothersome		
	To Treat	Not to Treat	To Treat	Not to Treat	To Treat	Not to Treat	To Treat	Not to Treat	To Treat	Not to Treat	Sign.
Image 1	12	31	78	101	65	57	34	5	0	0	χ^2^(3, *N* = 383) = 33.380, *p* = 0.000; τ_b_ = −0.245, *p* = 0.000
Image 2	4	4	63	39	94	47	114	18	0	0	χ^2^(3, *N* = 383) = 22.614, *p* = 0.000; τ_b_ = −0.218, *p* = 0.000
Image 3	4	3	22	11	54	18	206	18	45	2	χ^2^(4, *N* = 383) = 33.441, *p* = 0.000; τ_b_ = −0.259, *p* = 0.000
Image 4	7	8	41	79	83	80	61	17	6	2	χ^2^(4, *N* = 383) = 40.152, *p* = 0.000; τ_b_ = −0.282, *p* = 0.000
Image 5	1	1	6	1	16	6	118	4	226	4	χ^2^(4, *N* = 383) = 42.254, *p* = 0.000; τ_b_ = −0.1987, *p* = 0.005
Image 6	1	1	23	4	18	4	127	4	198	3	χ^2^(4, *N* = 383) = 32.934, *p* = 0.000; τ_b_ = −0.190, *p* = 0.003
Image 7	1	3	25	9	50	18	186	12	76	3	χ^2^(4, *N* = 383) = 7.749, *p* = 0.000; τ_b_ = −0.270, *p* = 0.000
Image 8	3	0	14	8	31	11	111	7	194	4	χ^2^(4, *N* = 383) = 54.523, *p* = 0.000; τ_b_ = −0.283, *p* = 0.000
Image 9	4	0	5	1	7	1	121	5	234	5	χ^2^(4, *N* = 383) = 7.206, *p* = 0.125; τ_b_ = −0.0878, *p* = 0.146

## Data Availability

The data presented in this study are available on request from the corresponding author.

## Appendix A

The paper-based questionnaire is presented in Figure A1.
